# Phycosphere microbiome contributes to ecological dominance of diatoms: a comparative study of *Cyclotella atomus* and *Ulnaria ulna*

**DOI:** 10.1093/ismeco/ycag211

**Published:** 2026-07-23

**Authors:** Gaofei Song, Fengfeng Cheng, Zhixian Qiao, Feng Ge, Yonghong Bi

**Affiliations:** Institute of Hydrobiology, Chinese Academy of Sciences, Wuhan 430072, Hubei, China; Institute of Hydrobiology, Chinese Academy of Sciences, Wuhan 430072, Hubei, China; Institute of Hydrobiology, Chinese Academy of Sciences, Wuhan 430072, Hubei, China; Institute of Hydrobiology, Chinese Academy of Sciences, Wuhan 430072, Hubei, China; Institute of Hydrobiology, Chinese Academy of Sciences, Wuhan 430072, Hubei, China

**Keywords:** diatom-bacteria interactions, ecological dominance, phycosphere microbiome, metabolic synergy, holobiont stability

## Abstract

Diatoms play a crucial role in aquatic ecosystems, yet the mechanisms underlying their long-term dominance remain poorly understood. This study investigated the relationship between diatom ecological persistence and their phycosphere bacterial communities by comparing the long-term dominant species *Cyclotella atomus* with the short-term dominant species *Ulnaria ulna*. 16S rRNA gene sequencing combined with predictive functional profiling revealed that the bacterial community associated with *C. atomus* was more diverse, stable, and interconnected than that associated with *U. ulna*. Taxonomic analysis identified key bacterial taxa such as *Gemmatimonas*, *Sphingobium*, and *Pseudorhodoferax* enriched in *C. atomus*. Co-occurrence network analysis demonstrated higher microbial interaction complexity in *C. atomus*, enhancing functional redundancy and ecosystem stability. Functional predictions indicated significant enrichment in carbohydrate metabolism (glycosaminoglycan degradation, pentose/glucose interconversion) and stress response pathways (betaine biosynthesis, xenobiotic metabolism by cytochrome P450) in the *C. atomus* microbiome, supporting a mutualistic relationship in which diatom-derived extracellular polymeric substances sustains specialized bacteria that reciprocate with vitamin B_12_, phytohormones, and chemical defenses. Based on these results, a mutually reinforced symbiotic cycle model was proposed to illustrate how the diatom and its phycosphere microbiome established a resilient holobiont capable of prolonged ecological dominance. The bacterial community associated with each diatom species exhibited host specificity and contributed to the maintenance of host dominance. These findings highlight the critical role of microbial partnerships in diatom success, offering new insights for predicting phytoplankton community dynamics and managing aquatic ecosystems.

## Introduction

Algae and bacteria engage in ubiquitous interactions ranging from symbiosis to parasitism, forming one of the most fundamental ecological linkages [1, 2]. These interactions transcend mere physical proximity, relying on sophisticated bidirectional chemical communication, material exchange, and signal transduction [3, 4]. Specific manifestations include coordinated nutrient exchange, mutual provision of growth factors, quorum sensing-mediated signaling, and occasional predation or lysis of partners for resource acquisition [5].

Among microalgae, diatoms represent a phylogenetically and ecologically pivotal group with unparalleled scientific and ecological significance. As globally dominant primary producers, they contribute ~20% of global primary production [6–8]. Their distinctive biosilica frustules not only mediate the marine silicon cycle but also enhance particulate organic carbon export via tight coupling between the silica pump and the biological carbon pump, thereby influencing global climate regulation [9]. The remarkable sensitivity of diatoms to environmental perturbations, combined with their relatively complete taxonomic system, establishes them as invaluable bioindicators for ecosystem monitoring [10]. Consequently, elucidating the mechanistic basis of diatom population dynamics, with particular emphasis on dominance maintenance, represents a fundamental challenge in understanding aquatic ecosystem resilience and function.

The temporal dynamics of diatom blooms, particularly the persistence of dominance phases, critically determine energy transfer efficiency through aquatic food webs and can trigger deleterious effects such as benthic hypoxia [11]. Yet, what determines whether a diatom species maintains long-term dominance or only transiently appears in the phytoplankton community remains incompletely understood. Traditional explanations have focused on abiotic factors (e.g. nutrient availability, light conditions, temperature) and intrinsic physiological traits (e.g. growth rates, nutrient uptake kinetics) in governing diatom population dynamics [12–15]. However, a potentially crucial player is the phycosphere, the immediate microenvironment surrounding diatom cells that harbors diverse and metabolically active bacterial communities.

Recent advances have substantially expanded our view of diatom-associated bacteria. The intricate microarchitecture of diatom cell surfaces, coupled with their secretion of diverse extracellular polymeric substances (EPS), creates highly specialized microhabitats that support complex epiphytic bacterial consortia [16, 17]. These phycosphere bacteria are not random assemblages; host-specific selection drives the assembly of distinct bacterial communities, with different diatom species or even strains harboring characteristic microbiota [18, 19]. Functionally, phycosphere bacteria can profoundly influence diatom physiology through multiple mechanisms. They provide essential vitamins (e.g. B_1_, B_7_, B₁₂), recycle nutrients (N, P, Fe), produce growth-promoting hormones (e.g. Indole-3-acetic acid, IAA), degrade harmful reactive oxygen species, and even produce allelopathic compounds that deter grazers or competing phytoplankton [19–26].

Despite these advances, most studies have focused on single diatom-bacterium pairs or short-term experiments under controlled conditions. A critical knowledge gap remains. How do phycosphere bacterial communities differ between diatom species with contrasting ecological strategies, and do these differences translate into distinct metabolic potentials that influence host persistence in the natural environment? To address this gap, we conducted a comparative analysis of the phycosphere microbiomes associated with two ecologically distinct diatom species isolated from the same habitat in the middle route of China’s South-to-North Water Diversion Project: *Cyclotella atomus*, which maintains dominance for several months, and *Ulnaria ulna*, which dominates for only a few weeks. Our study was designed with three objectives. First, to characterize and compare the community composition, diversity, and co-occurrence patterns of phycosphere bacteria between the two diatoms. Second, to predict the functional profiles and key enzymatic pathways of these bacterial communities using PICRUSt2, and to identify differentially enriched metabolic features. Third, to examine how the observed compositional and functional differences may contribute to the contrasting dominance persistence of the two diatom hosts. We therefore tested the hypothesis that differences in phycosphere community composition and metabolic potential are among the factors contributing to the contrasting dominance patterns of the two diatom hosts. By integrating community profiling with predictive functional inference, this study aims to provide novel mechanistic insights into the role of phycosphere bacteria in mediating diatom ecological success and to establish a foundation for understanding diatom–bacteria holobionts in aquatic ecosystems.

## Materials and methods

### Sample collection and DNA extraction

Two dominant diatom strains were isolated in June 2019 from the middle route of China’s South-to-North Water Diversion Project using a lab-made Pasteur pipette under a microscope (Olympus CX31, Japan). Morphological and molecular characterization identified them as *C. atomus* AEW011 (the long-term dominant species) and *U. ulna* AEW020 (the short-term dominant species). These two species represented the dominant phytoplankton taxa in the same waterway: *C. atomus* AEW011 maintained dominance from February to August 2019, while *U. ulna* AEW020 dominated from May to July 2019. For standardized cultivation, both strains were maintained in sterile CSI medium at 20°C under a 12:12 h light:dark cycle with a photon flux density of 35 μmol·m^−2^·s^−1^ provided by white fluorescent lamps.

To separate the diatoms and their associated phycosphere microbiomes from free-living bacteria in the culture medium, algal suspensions were filtered through sterile 3 μm polycarbonate membrane filters under gentle vacuum pressure (<5 psi). After filtration, the retained cells were gently rinsed once with 20 mL of sterile CSI medium to remove non-adherent bacteria. The retained biomass (diatoms with tightly associated epibionts) was immediately processed for downstream analyses.

The algal samples treated above (*C. atomus* AEW011 and *U. ulna* AEW020) were subjected to DNA extraction using the FastDNA® Spin Kit for Soil (MP Biomedical, Solon, OH, USA) according to the manufacturer’s instructions. DNA quality was assessed by running the extracted DNA on a 1.0% agarose gel and DNA concentrations were measured using a NanoDrop 1000 spectrophotometer (Thermo Scientific). Three biological replicates per species were collected for 16S rRNA gene amplicon sequencing.

### 16S rRNA gene amplicon sequencing

For targeted metabarcoding sequencing, the bacterial 16S rRNA gene V3-V4 region was amplified using the primer pair 341F (5’-CCTACGGGNGGCWGCAG-3′) and 805R (5’-GACTACHVGGGTATCTAATCC-3′) [27]. The polymerase chain reaction (PCR) was performed for each sample under the following conditions: 30 s at 95°C; 25 cycles of denaturation at 95°C for 30 s, annealing at 55°C for 30 s, and extension at 72°C for 30 s; followed by a final extension at 72°C for 5 min. The 25 μL of PCR mixture contained 12.5 μL of 2× KAPA HiFi HotStart ReadyMix (Roche, Switzerland), 5 μL of each primer (1 μM), and 2.5 μL of microbial DNA (5 ng/μL). The PCR products were electrophoresed and visualized by ethidium bromide staining. Specific DNA bands were excised and purified using AMPure XP beads (Beckman Coulter, USA). Sequencing libraries were generated using the Nextera XT Index Kit (Illumina, USA) following the manufacturer’s recommendations, and then paired-end (2 × 250 bp) sequencing was performed on the Illumina NovaSeq 6000 Sequencer (Illumina, Inc., CA, USA).

### 16S rRNA gene sequencing data processing and network analysis

For metabarcoding data, the reads were analyzed using QIIME 2 (Quantitative Insights Into Microbial Ecology 2, version 2019.7) and its plugins [28]. To obtain amplicon sequence variants (ASVs), we first used DADA2 (v1.18.0) to denoise the reads, remove low-quality sequences, and remove chimeras. Chimera removal was performed using the removeBimeraDenovo function with method = “consensus,” where a sequence is flagged as chimeric only if a majority of samples support that classification [29]. This step was applied after paired-end merging and prior to downstream filtering.

ASVs were then assigned to taxonomy using the QIIME2 feature-classifier plugin with a naïve Bayes classifier trained on the SILVA 138 database (targeting the V3–V4 region, corresponding to primers 341F–805R) at a confidence threshold of 0.7 [30, 31]. After taxonomic assignment, ASVs annotated as chloroplast, mitochondria, or k__Viridiplantae (plant-origin sequences) were removed using `qiime taxa filter-table` and `qiime taxa filter-seqs` with the —p-exclude parameter. This filtering step eliminates host-derived plastid 16S rRNA sequences that co-amplify with universal bacterial primers.

The DADA2 algorithm resolves ASVs at single-nucleotide resolution, capable of distinguishing sequences that differ by as little as 1 nucleotide, in contrast to traditional Operational Taxonomic Unit (OTU) clustering that groups sequences at a fixed 97% similarity threshold [29]. This high resolution enables accurate differentiation of closely related bacterial lineages.

Predicted functional characteristics of bacterial communities were obtained from the MetaCyc (https://metacyc.org/) and KEGG (https://www.kegg.jp/) database via PICRUSt2 (Version 2.2.0; https://github.com/picrust/picrust2), which predicts functional gene abundances based on the proportion of marker gene sequences in each sample [32]. Alpha diversity indices (Chao 1, Shannon, and Simpson’s Evenness) and beta diversity metrics (Bray-Curtis) were calculated using R v4.0.2 with the vegan package (v2.6–2) [33]. The relative abundances of KEGG Pathway level 3 and associated enzymes were visualized using Graphpad Prism (Version 9). The raw sequencing data have been deposited in the NCBI BioProject under the accession number PRJNA1429054.

For network analyses, co-occurrence networks were constructed using Spearman’s correlations calculated in R (version 4.0.2) with the Hmisc package (rcorr function) to assess interaction complexity within the phycosphere microbiome of each diatom species. The significance of local similarity scores (LS) was based on 1000 permutations. Only robust (LS > 0.6 or LS < −0.6) and statistically significant (*P* < .01) correlations were incorporated into network analyses [34]. The networks were visualized in Cytoscape v3.7.2 [35]. Subnetworks were extracted from the entire association network. The topological parameters of networks were calculated using the Network Analyzer in Cytoscape v.3.7.2, treating edges as undirected.

## Results

### Phycosphere bacterial community structure

A total of 652 164 sequences from six algal samples were obtained. After denoising and removing low-quality and chimeric sequences with DADA2, 439 amplicon sequence variants (ASVs) of epiphytic bacteria were retained.

**Figure 1:**
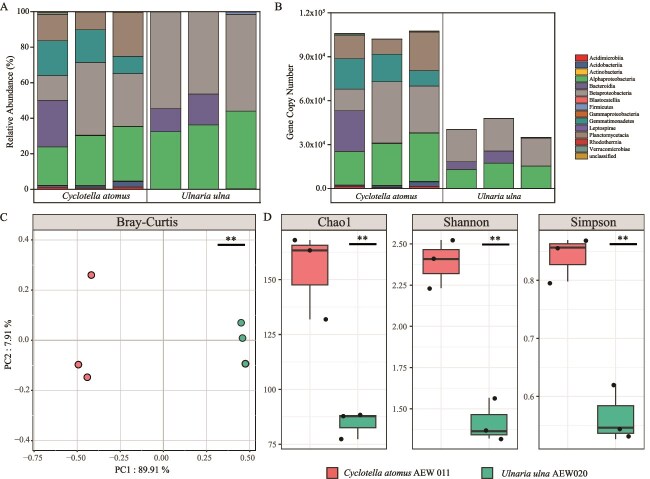
Community structure and diversity of the phycosphere bacterial community associated with *Cyclotella atomus* and *Ulnaria ulna*. (A) Relative abundance of bacterial taxa at the class level. (B) Gene copy number of bacterial taxa at the class level. (C) Principal coordinates analysis (PCoA) based on Bray-Curtis distances showing beta diversity differences between the two diatom species, with significance tested by ANOSIM. (D) Alpha diversity indices (Chao1 richness, observed ASVs, and Pielou’s evenness) of the phycosphere bacterial communities. Asterisks denote significant differences (*P < .05; **P < .01, ANOVA).

Results showed that the bacterial communities within the phycosphere exhibited distinct patterns of community structure and microbial diversity between *C. atomus* and *U. ulna* (Fig. 1). Principal coordinates analysis indicated that community structure differed significantly between the two diatoms (Analysis of Similarities, ANOSIM, *P* < .01). *C. atomus* harbored a phycosphere microbiome with greater microbial richness and evenness (Analysis of Variance, ANOVA, *P* < .01) than *U. ulna*.

**Figure 2:**
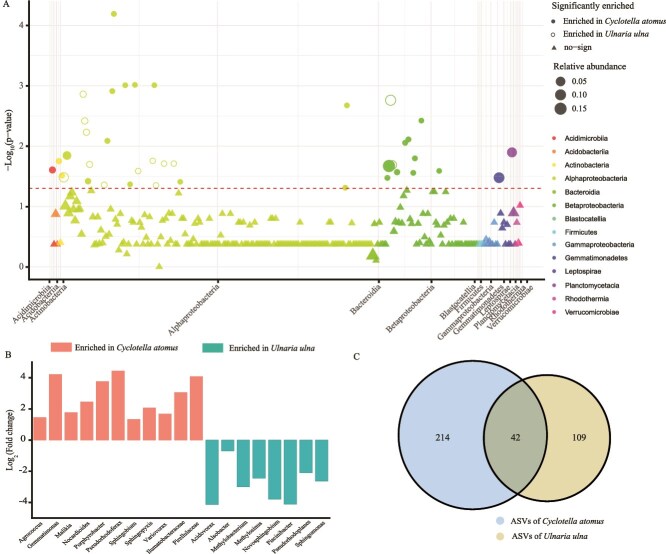
Taxonomic characteristics of differentially abundant bacteria in the phycosphere microbiomes of *Cyclotella atomus* and *Ulnaria ulna*. (A) Manhattan plots showing ASVs enriched in *C. atomus* (left) and *U. ulna* (right). (B) Genera significantly enriched in each diatom species (relative abundance >0.1%, *P* < .05). Bar length represents log_2_-transformed fold change. (C) Venn diagram depicting the number of ASVs identified in *C. atomus* and *U. ulna*.

Enrichment analysis revealed that the number of enriched ASVs in *C. atomus* was slightly higher than that in *U. ulna* (Fig. 2A). This was consistent with the results of the analysis of variance at the genus level (Fig. 2B). Overall, 26 and 13 bacterial ASVs were enriched in *C. atomus* and *U. ulna*, respectively*.* Among them, *Acidovorax* ASV3, *Gemmatimonas* ASV4, *Methylobacterium* ASV27, *Novosphingobium* ASV9, *Piscinibacter* ASV6, *Porphyrobacter* ASV11, *Pseudorhodoferax* ASV2, *Sphingobium* ASV104, *Sphingopyxis* ASV54, and *Pirellulaceae* ASV7 represented the “abundant variance bacteria” in the two diatoms (abundance >0.1%).

### Diatom-bacteria associations

**Figure 3:**
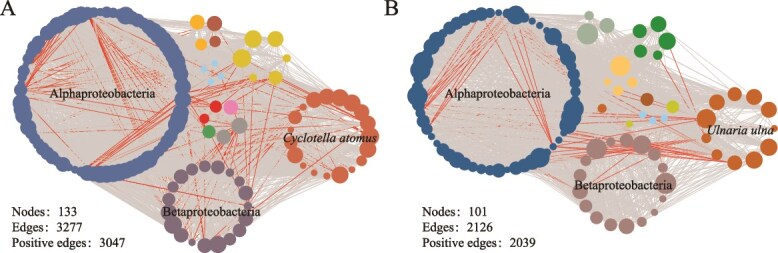
Co-occurrence networks of the phycosphere microbiomes associated with *Cyclotella atomus* and *Ulnaria ulna*. Networks show significant correlations (Spearman’s |r| > 0.6, *P* < .01) between bacterial ASVs. Each node represents a single ASV, colored by class-level taxonomy. Gray edges indicate positive correlations; red edges indicate negative correlations. (A) *C. atomus* phycosphere microbiome. (B) *U. ulna* phycosphere microbiome.

To explore whether differences in the phycosphere microbiome translate into distinct functional support for the diatom host, co-occurrence network analysis was performed. This analysis evaluates topological properties that reflect the degree of coordination and potential metabolic coupling among phycosphere bacteria, which in turn may influence the ecological services provided to the diatom. Co-occurrence network analysis revealed greater connectivity and complexity in the phycosphere microbiome of *C. atomus* (Fig. 3). The network of *C. atomus* presented a higher number of connections per node (average degree = 47.84) than that of *U. ulna* (40.89), indicating a highly interconnected bacterial community that may better support host persistence.

To examine the specific associations between the diatom host and its phycosphere bacteria, we performed Spearman’s correlation analysis between the relative abundance of *C. atomus* and the abundances of individual bacterial ASVs. A total of 839 significant correlations were identified (|r| > 0.6, *P* < .01), of which 765 (91.18%) were positive and 74 (8.82%) were negative (Table S1). Among genera with a relative abundance >0.01%, those positively correlated included *Altererythrobacter*, *Bosea*, *Devosia*, *Fimbriiglobus*, *Malikia*, *Methyloversatilis*, *Nocardioides*, *Novosphingobium*, *Opitutus*, *Paludibaculum*, *Reyranella*, *Sphingobium*, *Sphingomonas*, and *Sphingopyxis* (Table S1). Genera exhibiting both positive and negative correlations included *Acidovorax*, *Agrococcus*, *Brevundimonas*, *Flavobacterium*, *Gemmatimonas*, *Methylosinus*, *Methylobacterium*, *Piscinibacter*, *Prosthecobacter*, and *Pseudorhodoferax*.

For *U. ulna*, a total of 446 significant correlations were identified (|r| > 0.6, *P* < .01), with 410 (91.93%) positive and 36 (8.07%) negative (Table S2). Among genera with a relative abundance >0.01%, exclusively positive associations were observed with *Acidovorax*, *Pseudorhodoplanes*, and *Sphingomonas*, while exclusively negative associations were observed with *Caulobacter*, *Flavobacterium*, *Methylobacterium*, *Novosphingobium*, *Piscinibacter*, *Porphyrobacter*, *Pseudorhodoferax*, *Rhodoferax*, *Roseomonas*, *Sphingobium*, and *Sphingopyxis* (Table S2).

### Functional pathways of the phycosphere microbiome

**Figure 4:**
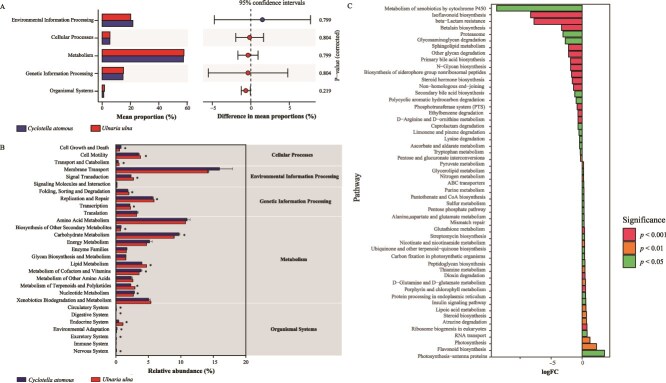
KEGG metabolic pathway classification of the phycosphere microbiomes associated with *Cyclotella atomus* and *Ulnaria ulna* at level 1 (A), level 2 (B), and level 3 (C). Values represent the mean percentage of sequences assigned to each functional category ± SEM. Asterisks indicate significant differences between species (*P < .05). logFC, log_2_-transformed fold change.

Differences in predicted metabolic pathways of the phycosphere microbiome were observed at multiple functional levels (Fig. 4). At KEGG pathway level 1 (Fig. 4A), no significant differences were detected between the two diatoms in the predicted functional categories of environmental information processing, cellular processes, metabolism, genetic information processing, and organismal systems (*P* > .05). At KEGG pathway level 2 (Fig. 4B), the abundances of cell growth and death, biosynthesis of other secondary metabolites, carbohydrate metabolism, metabolism of cofactors and vitamins nucleotide metabolism, environmental adaptation, and the nervous system were significantly higher in the phycosphere microbiome of *C. atomus* (*P* < .05), whereas the abundances of cell motility, signal transduction, folding/sorting/degradation, replication and repair, transcription, lipid metabolism, terpenoid and polyketide metabolism, and the circulatory, digestive, endocrine, and excretory systems were significantly higher in *U. ulna* (*P* < .05). At KEGG pathway level 3 (Fig. 4C), the figure presents the functional potential of the epiphytic bacteria community. In *C. atomus*, pathways such as xenobiotic metabolism by cytochrome P450, isoflavonoid biosynthesis, betalain biosynthesis, glycosaminoglycan degradation, N-glycan biosynthesis, tryptophan metabolism, and pentose/glucuronate interconversions were upregulated; in *U. ulna*, pathways such as photosynthesis, thiamine metabolism, and nitrogen metabolism were upregulated.

### Metabolic strategies

**Figure 5:**
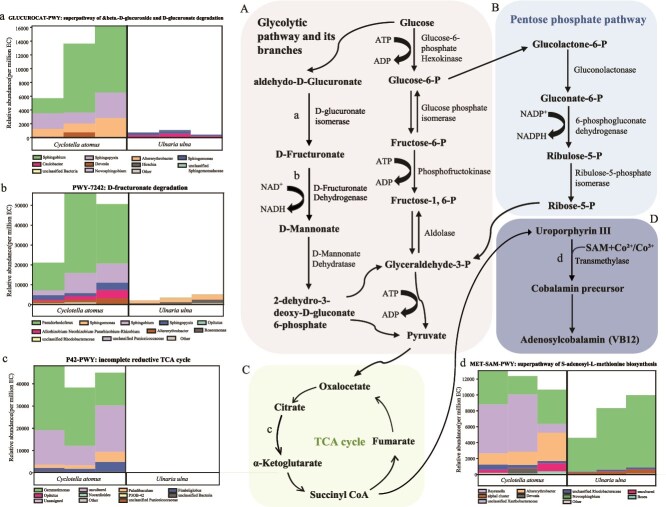
Predicted metabolic pathways and associated enzymes of the phycosphere microbiomes associated with *Cyclotella atomus* and *Ulnaria ulna*. The schematic illustrates four core metabolic modules: (A) glycolysis and its branch pathways, (B) the pentose phosphate pathway, (C) the tricarboxylic acid (TCA) cycle, and (D) vitamin B₁₂ biosynthesis-related compounds. Numbers a–d denote specific metabolic steps or enzymes whose predicted abundances differed significantly between the two phycosphere microbiomes (as inferred by PICRUSt2). Specifically: a, β-D-glucuronidase / D-glucuronate isomerase (involved in β-D-glucuronide and D-glucuronate degradation); b, D-fructuronate degradase (catalyzing D-fructuronate degradation to D-mannonate); c, enzymes of the incomplete reductive TCA cycle (bypassing α-ketoglutarate dehydrogenase); d, enzymes of the L-methionine biosynthesis I pathway (contributing to cobalamin precursor assembly).

Bacteria within the phycosphere of *C. atomus* exhibited significantly higher predicted enzymatic activities in the pathways such as β-D-glucuronide and D-glucuronate degradation, D-fructuronate degradation, the incomplete reductive TCA cycle, and L-methionine biosynthesis I (Fig. 5). These enzymes are distributed across core metabolic pathways, including glycolysis and its branch pathways, the pentose phosphate pathway, and the tricarboxylic acid (TCA) cycle. Their abundance variations are closely associated with vitamin B₁₂ (VB₁₂) synthesis.

## Discussion

By comparing the long-term dominant species *C. atomus* and the short-term dominant species *U. ulna*, significant differences in their phycosphere microbiomes were revealed, suggesting that the diatom-bacterial holobiont, rather than the diatom alone, may serve as the fundamental functional unit driving ecological performance [36, 37]. The underlying mechanisms likely involve host-mediated selection of specific bacterial partners through differential secretion of EPS and nutrients, which in turn creates a positive feedback loop that reinforces microbial community structure and metabolic capacity. Specifically, the phycosphere microbiome of the long-dominant *C. atomus* exhibited higher diversity, network complexity, and enrichment of specialized metabolic pathways (e.g. vitamin B₁₂ synthesis, carbohydrate degradation). These microbial traits may actively contribute to host persistence by enhancing nutrient acquisition, stress tolerance, and chemical defense against competitors. Thus, the observed differences in dominance duration cannot be fully explained by diatom physiology alone; instead, they appear to arise from the integrated functioning of the diatom and its associated bacterial community, with phycosphere bacteria playing a potentially causal role in extending host dominance.

Regarding community structure, the phycosphere microbiome of *C. atomus* was more diverse and evenly distributed than that of *U. ulna* (Fig. 1D). The higher richness (Chao 1 index) and evenness (Simpson index) indicated a more mature and stable microbial ecosystem, consistent with the ecological principle that stable host-microbe associations promote community complexity [36, 38, 39]. Such stability directly benefits the host by enhancing the ecosystem’s buffering capacity [40]. A diverse and even community provides greater functional redundancy; if environmental stress suppresses one bacterial group, others with similar metabolic roles can maintain critical services for diatom [41]. In contrast, the simpler and less stable community of *U. ulna* offers less functional redundancy, rendering the holobiont more vulnerable to perturbation, which may explain its transient dominance.

Taxonomic analysis revealed distinct enrichment patterns between the two diatoms (Fig. 2). *C. atomus* was significantly enriched with bacterial taxa including *Gemmatimonas* ASV4, *Porphyrobacter* ASV11, *Pseudorhodoferax* ASV2, *Sphingopyxis* ASV54, *Sphingobium* ASV104, and *Pirellulaceae* ASV7. These taxa are recognized for their metabolic versatility, involvement in nutrient cycling, environmental adaptation, and secretion of growth-promoting factors [3, 23, 42–46]. The enrichment of these multifunctional taxa likely equips *C. atomus* with a broader toolkit for resource acquisition and stress resilience compared to *U. ulna*.

Co-occurrence network analysis provided further ecological insights into how phycosphere bacterial assemblages may support the diatom host. The phycosphere microbiome of *C. atomus* exhibited higher connectivity and network complexity than that of *U. ulna* (Fig. 3), aligning with classical ecological theories that complex interaction networks enhance ecosystem stability and functional robustness [47]. A densely connected network suggests strong synergistic or cross-feeding interactions among bacteria. Such network properties may enhance functional redundancy and environmental adaptability, which could allow the holobiont to maintain functional integrity under environmental disturbances [48, 49]. Conversely, the sparser network of *U. ulna* implies a more loosely associated assemblage, which is potentially less coordinated and more susceptible to collapse under stress, contributing to its shorter dominance periods. Thus, the more interconnected bacterial community associated with *C. atomus* likely translates into more reliable delivery of host-beneficial services, supporting its long-term ecological persistence.

Beyond structural differences, predicted metabolic functions provided crucial mechanistic insights. The bacterial community associated with *U. ulna* showed enrichment in fundamental pathways such as photosynthesis and nitrogen metabolism, suggesting a relatively casual and loosely integrated relationship in which bacteria primarily benefit from the phycosphere niche. In contrast, the microbiome of *C. atomus* displayed distinct enrichment in specialized metabolic pathways. The significant upregulation of pathways involved in degrading complex carbohydrates, such as glycosaminoglycan degradation and the interconversion of pentoses and glucose, strongly implies a co-evolutionary relationship centered on carbon cycling [50–52]. Previous studies suggest that algal–bacterial interactions involving EPS and antioxidant production enhance survival under stress [53]. *C. atomus* likely secretes specific, complex EPS in response to challenging conditions, thereby selecting for specialized bacterial taxa capable of efficiently utilizing these polymers [54–57]. Enrichment in betaine biosynthesis and tryptophan metabolism pathways among the *C. atomus* phycosphere microbiome was also notable. Betaine serves as a multifunctional metabolite that protects cells from drought, osmotic, and thermal stress, while also enhancing the performance of microbial strains involved in the synthesis of vitamin B₁₂, lactate, ethanol, lysine, and pyruvate [58]. Moreover, many microalgae are auxotrophic for vitamins, phytohormones, thiamine derivatives, and siderophores [59]. The phytohormone IAA plays an important role in promoting algal growth, and Zhang et al. [60] reported that 82.2% of analyzed bacterial genomes harbor the potential to synthesize IAA from tryptophan or its intermediates. Upregulation of these pathways is thus critical for stimulating diatom growth and sustaining positive interkingdom interactions. Additionally, enrichment in pathways such as isoflavonoid biosynthesis, β-lactam resistance, and cytochrome P450-mediated xenobiotic metabolism points to enhanced capacities in anti-inflammatory, antipathogenic, antioxidant, biodegradative, and drug-metabolizing functions. Such chemical defenses may aid the host in detoxifying harmful compounds or producing allelochemicals to suppress competing algae [44, 61, 62]. This process enables direct remineralization of organic matter within the phycosphere, establishing a tight and efficient nutrient loop that, together with functional metabolites and chemical defenses, minimizes resource leakage to the surrounding environment and competing phytoplankton.

Differences in enzymatic potential among microbial communities lead to varied functional contributions to specific metabolic end products. The initial step of the superpathway for β-D-glucuronide and D-glucuronate degradation involves hydrolysis by β-D-glucuronidase to yield D-glucuronate, which is then isomerized to D-fructuronate by D-glucuronate isomerase. D-fructuronate is subsequently converted to D-mannonate by D-fructuronate dehydratase [63]. Compared with *U. ulna*, *C. atomus* showed higher abundances of enzymes involved in this degradation superpathway as well as D-fructuronate dehydratase, indicating stronger metabolic flux through glycolytic branch routes. Under these conditions, glycolysis can generate more D-glyceraldehyde-3-phosphate and pyruvate. Increased D-glyceraldehyde-3-phosphate drives more glucose-6-phosphate into the pentose phosphate pathway while also entering the tricarboxylic acid (TCA) cycle along with pyruvate. In the TCA cycle, enzymes associated with the incomplete reductive TCA cycle bypass the oxidative decarboxylation catalyzed by the α-ketoglutarate dehydrogenase complex, preserving intermediates such as α-ketoglutarate and succinyl-CoA that are preferentially directed toward the synthesis of amino acids, porphyrin rings, and nucleotides [64]. Uroporphyrinogen III is chelated with cobalt via enzymes of the L-methionine biosynthesis I pathway to form cobalamin precursors [45, 65]. The abundances of enzymes related to the incomplete reductive TCA cycle and the L-methionine biosynthesis I pathway were significantly higher in *C. atomus* than in *U. ulna*. Integrating these metabolic and enzymatic patterns, we infer that D-glyceraldehyde-3-phosphate and pyruvate produced through glycolysis and its branches can both supply ATP for vitamin B₁₂ synthesis and channel more glucose-6-phosphate into the pentose phosphate pathway to generate NADPH, providing reducing equivalents for corrin ring modification. The pentose phosphate pathway also contributes 5-phosphoribose, which participates in purine/pyrimidine synthesis for vitamin B₁₂ assembly. Enriched succinyl-CoA from the incomplete reductive TCA cycle directly feeds into δ-aminolevulinic acid (ALA) synthesis, furnishing the carbon skeleton for vitamin B₁₂. During cobalamin assembly, S-adenosylmethionine donates methyl groups for corrin ring methylation, ultimately yielding adenosylcobalamin, the active form of vitamin B₁₂, via specific enzymes [66]. The taxonomic composition of these four key enzyme-differential pathways corresponded with genus level enrichment results. Cobalamin (vitamin B₁₂) plays essential roles in algal amino acid and carbon metabolism; however, over 50% of microalgae are auxotrophic for this cofactor and must acquire it exogenously or through bacterial partners [22, 46]. Thus, the phycosphere microbiome of *C. atomus* could reliably provide this critical growth factor, conferring a strong competitive advantage to planktonic *C. atomus*, particularly under nutrient-limiting conditions.

We acknowledge that free-living bacterial communities were not characterized; therefore, we cannot determine whether the metabolic pathways enriched in the phycosphere microbiomes of *C. atomus* and *U. ulna* are host-specific or also prevalent in free-living bacterioplankton. Because our diatom strains were isolated and subsequently cultured in the laboratory, the phycosphere communities analyzed may not fully recapitulate in situ diatom–bacteria interactions. This limitation is inherent to the source water body (the middle route of China’s South-to-North Water Diversion Project), which is oligotrophic to mesotrophic with rare algal blooms; moreover, its management requirements precluded the use of classical eutrophic culturing approaches to obtain sufficient biomass of both target diatoms while preserving their native bacterial communities. Nevertheless, our primary objective was to compare the functional potential of bacteria intimately associated with two diatom hosts exhibiting markedly different dominance patterns. We therefore focused on the attached phycosphere fraction, which is more likely to mediate host-specific ecological benefits. Importantly, a growing body of evidence indicates that algal host traits, together with the initial microbial inoculum, exert stronger influences on phycosphere community composition than free-living bacteria [18, 67]. Future studies integrating parallel sequencing of both the phycosphere and free-living fractions from the same environmental samples, ideally collected across different dominance stages, are needed to validate our proposed mutually reinforced symbiotic cycle model under natural conditions.

## Conclusion

This study suggests that the diatom and its specialized phycosphere microbiome may establish a stable symbiotic relationship through positive feedback. The host-derived EPS appears to shape a distinct bacterial assemblage, while the bacteria may enhance host fitness through multiple functional services, including organic matter recycling, growth factor provision, and chemical defense. This synergy could render the diatom-bacteria holobiont a highly adaptable and competitive ecological unit. While our findings support the hypothesis that the microbiome contributes to diatom ecological strategies, the conclusions remain correlative due to the lack of direct experimental validation and the use of laboratory isolates. The success of *C. atomus* may depend not only on its intrinsic traits but also on its co-evolved symbiotic relationships with its specific phycosphere microbiome. Therefore, we propose the mutually reinforced symbiotic cycle as a working model that requires further testing under natural conditions using integrated metagenomic, transcriptomic, and co-culture approaches. These findings offer fresh perspectives for predicting and managing algal blooms, emphasizing the need for an integrative approach that considers host-microbiome interactions in ecological research, while acknowledging that causality remains to be established.

## Supplementary Material

Supplementary_material_ycag211

## Data Availability

The raw metagenomic sequencing data supporting the findings of this study have been deposited in the NCBI [GenBank] repository under the accession numbers PRJNA1429054 (http://www.ncbi.nlm.nih.gov/bioproject/1429054).
