# Supplementary material for: Phycosphere microbiome contributes to ecological dominance of diatoms: a comparative study of Cyclotella atomus and Ulnaria ulna
Source: ISME Commun. 2026 Jul 23;6(1):ycag211. doi: 10.1093/ismeco/ycag211 (PMC13477216; doi:10.1093/ismeco/ycag211)
Supplement: Supplementary file 1 [file supplementary_material_ycag211.docx]

**Table legends:**

**Tab. S1** Bacterial genera with relative abundance >0.01% showing significant correlations with *Cyclotella atomus* (Spearman’s |r| > 0.6, P < 0.01)

**Tab. S2** Bacterial genera with relative abundance >0.01% showing significant correlations with *Ulnaria ulna* (Spearman’s |r| > 0.6, P < 0.01)

Table S1 Bacterial genera with relative abundance >0.01% showing significant correlations with *Cyclotella atomus* (Spearman’s |r| > 0.6, *P* < 0.01)

| *Cyclotella atomus* | phycosphere bacteria | correlation |
| --- | --- | --- |
| ASV43 | *Acidovorax* ASV58 | 0.92632 |
| ASV43 | *Acidovorax* ASV66 | 0.92632 |
| ASV57 | *Acidovorax* ASV58 | 1.00000 |
| ASV57 | *Acidovorax* ASV66 | 1.00000 |
| ASV8 | *Acidovorax* ASV58 | -0.79021 |
| ASV8 | *Acidovorax* ASV66 | -0.79021 |
| ASV81 | *Acidovorax* ASV58 | 0.99825 |
| ASV81 | *Acidovorax* ASV66 | 0.99825 |
| ASV1 | *Agrococcus* ASV80 | -0.64448 |
| ASV82 | *Agrococcus* ASV80 | 0.99121 |
| ASV1 | Allorhizobium-Neorhizobium-Pararhizobium-Rhizobium ASV18 | 1.00000 |
| ASV1 | *Altererythrobacter* ASV19 | 1.00000 |
| ASV1 | *Bosea* ASV46 | 1.00000 |
| ASV1 | *Brevundimonas* ASV53 | -0.76708 |
| ASV82 | *Brevundimonas* ASV53 | 0.92443 |
| ASV43 | *Devosia* ASV42 | 0.98234 |
| ASV57 | *Devosia* ASV42 | 0.85213 |
| ASV81 | *Devosia* ASV42 | 0.87479 |
| ASV56 | *Fimbriiglobus* ASV12 | 0.92632 |
| ASV1 | *Flavobacterium* ASV5 | -0.79021 |
| ASV82 | *Flavobacterium* ASV5 | 0.92632 |
| ASV1 | *Gemmatimonas* ASV4 | -0.79021 |
| ASV1 | *Gemmatimonas* ASV63 | -0.79021 |
| ASV82 | *Gemmatimonas* ASV4 | 0.92632 |
| ASV82 | *Gemmatimonas* ASV63 | 0.92632 |
| ASV1 | *Malikia* ASV59 | 1.00000 |
| ASV43 | *Methylobacterium* ASV32 | 0.92632 |
| ASV43 | *Methylobacterium* ASV44 | 0.92632 |
| ASV57 | *Methylobacterium* ASV32 | 1.00000 |
| ASV57 | *Methylobacterium* ASV44 | 1.00000 |
| ASV8 | *Methylobacterium* ASV32 | -0.79021 |
| ASV8 | *Methylobacterium* ASV44 | -0.79021 |
| ASV81 | *Methylobacterium* ASV32 | 0.99825 |
| ASV81 | *Methylobacterium* ASV44 | 0.99825 |
| ASV43 | *Methylosinus* ASV74 | 0.98067 |
| ASV57 | *Methylosinus* ASV74 | 0.97023 |
| ASV8 | *Methylosinus* ASV74 | -0.68301 |
| ASV81 | *Methylosinus* ASV74 | 0.98070 |
| ASV43 | *Methyloversatilis* ASV51 | 0.75790 |
| ASV82 | *Methyloversatilis* ASV51 | 0.75790 |
| ASV1 | *Nocardioides* ASV36 | 1.00000 |
| ASV56 | *Novosphingobium* ASV9 | 0.92632 |
| ASV56 | *Opitutus* ASV23 | 0.92443 |
| ASV1 | *Paludibaculum* ASV14 | 1.00000 |
| ASV43 | *Piscinibacter* ASV6 | 0.94376 |
| ASV43 | *Piscinibacter* ASV70 | 0.92443 |
| ASV57 | *Piscinibacter* ASV6 | 0.99825 |
| ASV57 | *Piscinibacter* ASV70 | 0.99825 |
| ASV8 | *Piscinibacter* ASV6 | -0.76708 |
| ASV8 | *Piscinibacter* ASV70 | -0.76708 |
| ASV81 | *Piscinibacter* ASV6 | 1.00000 |
| ASV81 | *Piscinibacter* ASV70 | 0.99649 |
| ASV56 | *Prosthecobacter* ASV11 | 0.75790 |
| ASV57 | *Prosthecobacter* ASV11 | -0.79021 |
| ASV8 | *Prosthecobacter* ASV11 | 1.00000 |
| ASV81 | *Prosthecobacter* ASV11 | -0.76708 |
| ASV56 | *Prosthecobacter* ASV20 | 0.75790 |
| ASV57 | *Prosthecobacter* ASV20 | -0.79021 |
| ASV8 | *Prosthecobacter* ASV20 | 1.00000 |
| ASV81 | *Prosthecobacter* ASV20 | -0.76708 |
| ASV43 | *Pseudorhodoferax* ASV2 | 0.92632 |
| ASV57 | *Pseudorhodoferax* ASV2 | 1.00000 |
| ASV8 | *Pseudorhodoferax* ASV2 | -0.79021 |
| ASV81 | *Pseudorhodoferax* ASV2 | 0.99825 |
| ASV43 | *Reyranella* ASV13 | 0.75790 |
| ASV82 | *Reyranella* ASV13 | 0.75790 |
| ASV43 | *Sphingobium* ASV10 | 0.92632 |
| ASV57 | *Sphingobium* ASV10 | 1.00000 |
| ASV81 | *Sphingobium* ASV10 | 0.99825 |
| ASV1 | *Sphingomonas* ASV104 | 0.60702 |
| ASV1 | *Sphingomonas* ASV122 | 0.92444 |
| ASV43 | *Sphingomonas* ASV122 | 0.60318 |
| ASV43 | *Sphingomonas* ASV15 | 0.98234 |
| ASV56 | *Sphingomonas* ASV104 | 0.92958 |
| ASV57 | *Sphingomonas* ASV15 | 0.85213 |
| ASV81 | *Sphingomonas* ASV122 | 0.73592 |
| ASV81 | *Sphingomonas* ASV15 | 0.87479 |
| ASV56 | *Sphingopyxis* ASV16 | 0.92632 |

Table S2 Bacterial genera with relative abundance >0.01% showing significant correlations with *Ulnaria ulna* (Spearman’s |r| > 0.6, *P* < 0.01)

| *Ulnaria ulna* | phycosphere bacteria | correlation |
| --- | --- | --- |
| ASV1 | *Acidovorax* ASV3 | 1.0000 |
| ASV43 | *Caulobacter* ASV38 | -0.7902 |
| ASV43 | *Flavobacterium* ASV5 | -0.7902 |
| ASV35 | *Methylobacterium* ASV27 | -0.7902 |
| ASV35 | *Methylobacterium* ASV28 | -0.7902 |
| ASV56 | *Methylobacterium* ASV27 | -0.6445 |
| ASV56 | *Methylobacterium* ASV28 | -0.6445 |
| ASV67 | *Methylobacterium* ASV27 | -0.7902 |
| ASV67 | *Methylobacterium* ASV28 | -0.7902 |
| ASV43 | *Novosphingobium* ASV9 | -0.7902 |
| ASV43 | *Piscinibacter* ASV6 | -0.7902 |
| ASV35 | *Porphyrobacter* ASV11 | -0.7902 |
| ASV67 | *Porphyrobacter* ASV11 | -0.7902 |
| ASV35 | *Pseudorhodoferax* ASV2 | -0.7902 |
| ASV56 | *Pseudorhodoferax* ASV2 | -0.6445 |
| ASV67 | *Pseudorhodoferax* ASV2 | -0.7902 |
| ASV35 | *Pseudorhodoplanes* ASV49 | 1.0000 |
| ASV56 | *Pseudorhodoplanes* ASV49 | 0.9527 |
| ASV67 | *Pseudorhodoplanes* ASV49 | 1.0000 |
| ASV35 | *Rhodoferax* ASV29 | -0.7902 |
| ASV56 | *Rhodoferax* ASV29 | -0.6445 |
| ASV67 | *Rhodoferax* ASV29 | -0.7902 |
| ASV43 | *Roseomonas* ASV22 | -0.7902 |
| ASV35 | *Sphingobium* ASV10 | -0.7902 |
| ASV56 | *Sphingobium* ASV10 | -0.6445 |
| ASV67 | *Sphingobium* ASV10 | -0.7902 |
| ASV35 | *Sphingomonas* ASV15 | 1.0000 |
| ASV56 | *Sphingomonas* ASV15 | 0.9527 |
| ASV67 | *Sphingomonas* ASV15 | 1.0000 |
| ASV35 | *Sphingopyxis* ASV16 | -0.7215 |
| ASV67 | *Sphingopyxis* ASV16 | -0.7215 |
